# Doxorubicin-enriched, ALDH^br^ mouse breast cancer stem cells are treatable to oncolytic herpes simplex virus type 1

**DOI:** 10.1186/1471-2407-12-549

**Published:** 2012-11-23

**Authors:** Xiufen Zhuang, Wen Zhang, Yatong Chen, Xiangping Han, Jie Li, Yu Zhang, Youhui Zhang, Shuren Zhang, Binlei Liu

**Affiliations:** 1 Department of Immunology, Cancer Institute & Hospital, Chinese Academy of Medical Sciences & Peking Union Medical College, No. 17 Panjiayuan Nanli Chaoyang District, Beijing, 100021, China; 2 Department of Urologic Surgery, Beijing Anzhen Hospital, Capital Medical University, Beijing, 100029, China; 3 Department of Biochemistry and Molecular Biology, College of Life Sciences, Nankai University, Tianjin, 300192, China; 4 Tianjin International Joint Academy of Biotechnology & Medicine, Tianjin, 300457, China

**Keywords:** Cancer stem cells, Breast cancer, Chemoresistant, ALDH, Oncolytic virus, Doxorubicin, Herpes simplex virus

## Abstract

**Background:**

The primary objective of this study was to test whether oncolytic herpes simplex virus type 1 (HSV1) could eradicate chemoresistant cancer stem cells (CSCs).

**Methods:**

The fluorescent aldefluor reagent-based technique was used to identify and isolate ALDH^br^ cells as CSCs from the 4T1 murine breast cancer cell line. The presence of ALDH^br^ 4T1 cells was also examined in 4T1 breast cancer transplanted in immune-competent syngeneic mice.

**Results:**

Compared with ALDH^lo^ cells, ALDH^br^ cells had a markedly higher ability to form tumor spheres *in vitro* and a higher tumorigenic potential *in vivo*. ALDH^br^ cells also exhibited increased doxorubicin resistance *in vitro*, which correlated with a selective increase in the percentage of ALDH^br^ cells after doxorubicin treatment and an increased expression of P-glycoprotein (P-gp), a known chemoresistance factor. In contrast, oncolytic HSV1 was able to kill ALDH^br^ cells *in vitro* and even more markedly *in vivo*. Furthermore, in *in vivo* studies, systemic administration of doxorubicin followed by intratumoral injection of oncolytic HSV1 resulted in much more significant suppression of tumor growth with increased median survival period compared with each treatment given alone (p<0.05). Though more CD8^+^ T lymphocytes were induced by oncolytic HSV1, no significant specific T cell response against CSCs was detected *in vivo*.

**Conclusions:**

These results suggested that the use of oncolytic HSV1 following doxorubicin treatment may help eradicate residual chemoresistant CSCs *in vivo*.

## Background

Breast cancer is the 3^rd^ most commonly diagnosed type of cancer and the leading cause of cancer death among females aged 20 to 59 years old [1]. With the improvement of early detection and/or comprehensive treatment, the breast cancer death rate has been greatly reduced. However, there are no effective therapeutic treatments once cancer is recurrent or metastatic. Cancer stem cells (CSCs) have been considered responsible for cancer progression, recurrence, metastasis and resistance to a number of conventional therapies [2-7]. CSCs have been identified in many tumor types and cell lines based on the expression of unique cell surface markers such as CD44, CD24 and CD133 or their ability to efflux lipophilic, fluorescent dyes such as Hoechst 33342 [8-11]. Another useful approach for the identification of CSCs is based on a high level of aldehyde dehydrogenase (ALDH^br^) activity, which has been associated with chemoresistance and poor prognosis in many types of cancer [12-18]. The 4T1 mouse breast cancer cell line was chosen for this study because its growth and metastatic spread in mice closely mimic stage IV human breast cancer [19]. Several markers, including Sca-1 and ALDH^br^, have been used for 4T1 CSC isolation in recent reports [20,21]. In this study, we used ALDH^br^ to identify and isolate CSCs from 4T1 cells. Because most conventional treatment regimens, including chemotherapy, target the non-CSC population of the tumor and fail to eliminate CSCs [5,6], discovering new ways to eliminate CSCs that are left behind following chemotherapy is important. The use of oncolytic viruses (OVs) is likely the treatment of choice [22-24]. OVs are either naturally occurring or genetically engineered viruses that selectively infect and lyse tumor cells without deleterious effects on normal cells [25,26]. In the OV family, the oncolytic herpes simplex virus type 1 (HSV1) is one of the most extensively studied, and it has strong oncolytic activity and promising therapeutic effects [27-29]. Given that the anticancer mechanism of viral oncolysis differs from that of chemotherapeutic agents, we reasoned that chemoresistant CSCs may be still treatable to oncolytic HSV1 [30-34]. In this study, we compared the role of oncolytic HSV1 and doxorubicin in the eradication of CSCs *in vitro* and *in vivo* and then employed doxorubicin to kill non-CSCs, followed by oncolytic HSV1 administration to eradicate residual chemoresistant CSCs *in vivo*. CD8^+^ cytotoxic T lymphocytes (CTLs) have been shown to play a critical role in immunity against cancer and viruses [35-37]. We therefore further investigated whether the immunological mechanism induced by our oncolytic HSV1 mediated the eradication of CSCs.

## Methods

### Cells and drugs

4T1, a mammary gland tumor cell line from Balb/c mice with high metastatic potency, was purchased from ATCC. 4T1 cells were maintained in DMEM/F12 supplemented with 10% FCS and gentamycin at 37°C in a humidified atmosphere of 5% CO_2_. The cells were subcultured at a ratio 1:6–8 when they were close to 80% confluent, approximately every 2 to 3 days. Cells of the 3^rd^-6^th^ passages in the log growth phase were used for experiments.

Doxorubicin, a classic chemotherapy agent for human breast cancers, was purchased from Shenzhen Main Luck Pharmaceuticals Inc. and prepared at 1.5 mg/ml in saline just prior to use.

### Construction of recombinant oncolytic HSV1-GFP and HSV1-hGM-CSF

Oncolytic HSV1-GFP and HSV1-hGM-CSF, constructed in our laboratory, were attenuated oncolytic herpes simplex type 1 viruses (17+, ECACC 0104151v). All virus vectors were grown in BHK-21 [C13] (ATCC: CCL-10) or Vero (ATCC: CCL-81) cells in DMEM/F12 containing 10% FCS. Viral DNA was purified using DNAzol (BioTeke Corporation, China).

To construct HSV1-GFP and HSV1-hGM-CSF, the genes encoding infection cell protein 34.5 (ICP34.5) and ICP47 were removed, the expression cassette for human granulocyte–macrophage colony-stimulating factor (hGM-CSF) or green fluorescent protein (GFP) was inserted into the sites for ICP34.5.

To delete ICP47, the up-stream (US) and down-stream (DS) flanking regions (FLRs) were amplified from 17+ virus genome with primer pairs ICP47USf versus ICP47USr and ICP47DSf versus ICP47DSr, respectively (Table 1). Then the *EcoRI*/*SpeI*-digested US and *HindIII*/*SalI*-digested DS FLRs were jointed together with the complemented Linker 1/2 (Table 1) and subsequently cloned into pBluescript (Stratagene) *EcoRI* and *SalI* sites to create pdICP47, which was then sequencing verified. The eGFP expression cassette under control CMV promoter released from pcDNA3.1-eGFP (YRGENE, China) by *EcoRI*/*XhoI* double digestion and treated with T4 DNA polymerase was cloned into pdICP47 *EcoRV* site to generate pdICP47-eGFP.

**Table 1:** The primers used for the construction of pdICP34.5 and pdICP47 are listed

**Primer name**	**Sequence**
ICP34.5USf	^151458^CTCTGACCTGAGATTGGCGGCACTG^151482^
ICP34.5USr	**GCGGCCGCAGCGCTGCGGCCGC**^644^CGCGGGCGCGCTCCTGACCGCGGG^621^
ICP34.5DSf	**GCGGCCGCAGCGCTGCGGCCGC**^1426^CAGCGCGGCGGGGCCCGGCCAACCA^1450^
ICP34.5DSr	^2895^TTCTTCCCTCTTCTCCCGCCCTCCA^2871^
ICP47USf	AAAA***GAATTC***GAT^143675^TGGGTTCGATTGGCAATGTTGTCTC^143699^
ICP47USr	AAAA***ACTAGT***GAT^145310^GTCCCGGGTACGACCATCACCCGAG^145286^
ICP47DSf	AAAA***AAGCTT***^145570^CACGACATGCTCCCCCCCGACGAGC^145594^
ICP47DSr	AAAA***CAGCTG***^146980^ACGCGGAACTAGCGCGGACCGGTCG^146956^
Linker 1	***CTAGT***GAATTCTAGTGGATCCCCCGGGCTGCAGGAATTCGATATC***A***
Linker 2	***AGCTT***GATATCGAATTCCTGCAGCCCGGGGGATCCACTAGAATTC***A***

The genome coordinates for the underlined genomic sequences are indicated. The restriction sites used for the construction of the shuttle plasmids are in bold italics. The reverse-complement sequences present in ICP34.5USr and ICP34.5DSf for overlapping PCR to join ICP34.5 US and DS FLRs are in bold.

**Figure 1:**
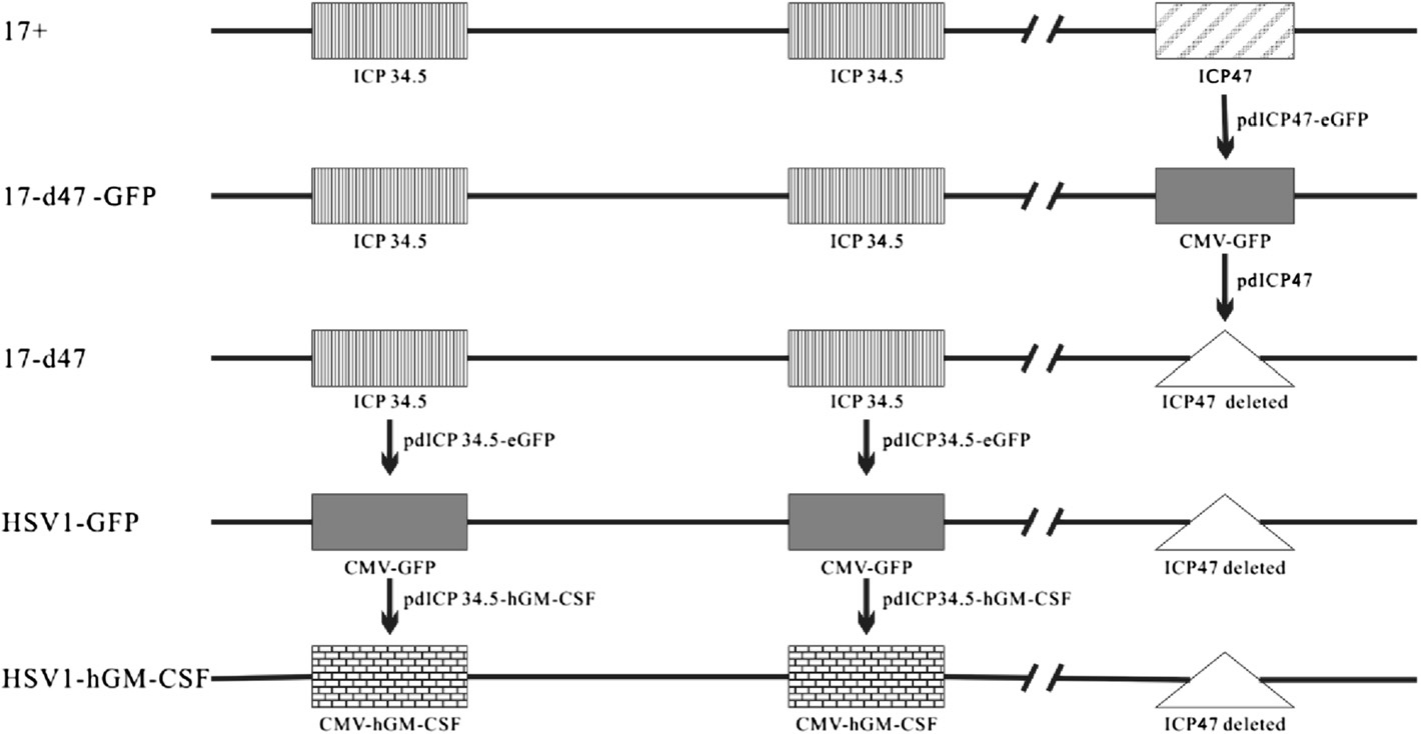
**Schematic construction of oncolytic HSV1-GFP and HSV1-hGM-CSF. **The two oncolytic HSV1 vectors were developed from 17+ strain. First, the ICP47 gene was removed from the virus genome by pdICP47-eGFP and pdICP47. Then, the GFP expression cassette from pdICP34.5-eGFP was inserted into both sites for ICP34.5 genes to generate HSV1-GFP. The hGM-CSF expression cassette from pdICP34.5-hGM-CSF was used to replace GFP expression cassettes in HSV1-GFP to create HSV1-hGM-CSF.

Upon the removal of ICP47, 17+ strain viral DNA and pdICP47-eGFP were co-transfected into BHK cells to allow homologous recombination. The recombined vector (17-d47-GFP) expressing eGFP was purified with four round plaque assays. At each round, 4–6 single plaques were picked under fluorescent microscope. With similar procedure, the 17-d47 vector (Figure 1) with the eGFP expression cassette removed was constructed by co-transfection of 17-d47-GFP viral DNA and pdICP47.

To delete ICP34.5, the US and DS FLRs were amplified from 17+ strain genome with primer pairs ICP34.5USf versus ICP34.5USr and ICP34.5DSf versus ICP34.5DSr, respectively (Table 1). Then the ICP34.5 US and DS FLRs were jointed together using an overlapping PCR with the primer pair ICP34.5USf/ICP34.5DSr and subsequently inserted into pSP72 (Promega) pre-digested with *BamHI*/*XhoI* and treated with T4 DNA polymerase for blunt-end cloning. The resulted plasmid was named as pdICP34.5 and sequencing verified. The hGM-CSF gene (Invivogen)was used to replace eGFP of pcDNA3.1-eGFP giving plasmid pcDNA3.1-hGM-CSF. The eGFP and hGM-CSF expression cassettes from pcDNA3.1-eGFP and pcDNA3.1- hGM-CSF were cloned into pdICP34.5 *AfeI* site to generate pdICP34.5-eGFP and pdICP34.5-hGM-CSF, respectively. The pdICP34.5-eGFP and pd.ICP34.5-hGM-CSF were used to delete ICP34.5 from 17-d47 vector giving viruses HSV1-GFP and HSV1-hGM-CSF (Figure 1).

### Flow cytometry sorting of cells with ALDH^br^ activity

4T1 cells were harvested, and a single-cell suspension was obtained for the aldefluor assay according to the manufacturer's instructions (Stem Cell Technologies). Briefly, 10^6^ cells were resuspended in 1 ml of aldefluor assay buffer containing activated aldefluor substrate. As a negative control for each sample, an aliquot of “aldefluor-exposed” cells was immediately quenched with a specific ALDH inhibitor, diethylaminobenzaldehyde (DEAB). Following a 30-minute incubation at 37°C, the cells were centrifuged, the pellets were resuspended in 0.5 ml aldefluor assay buffer, and the ALDH^br^ and ALDH^lo^ subpopulations were sorted using a FACSDiVa flow cytometer (Becton Dickinson).

### Mammosphere formation assay

4T1 or isolated cells were resuspended in DMEM/F12 serum free medium (SFM) supplemented with human recombinant epidermal growth factor (EGF; 20 ng/ml) and basic fibroblast growth factor (bFGF; 20 ng/ml) and then seeded in ultra-low attachment 6-well plates (Costar, Corning Incorporated) with 5 × 10^4^ cells/well in 2 ml. Both EGF and bFGF were purchased from Sigma Biochemicals. Fresh aliquots of EGF and bFGF were added every other day. After 8 days of culture, mammospheres were observed. Carboxymethyl cellulose (CMC) was added at a final concentration of 0.8% to keep the fluid flow slow, and the spheres were quantified using an inverted phase contrast microscope (Olympus Co.).

### Tumorigenicity studies with isolated cells

The sorted ALDH^br^ and ALDH^lo^ cells were resuspended, serially diluted in DMEM/F12 SFM and inoculated subcutaneously (s.c.) into the right flanks of 6-7-week-old immune-competent female Balb/c mice (n=5-6) at varying numbers (5, 000, 1, 000 and 100) in a volume of 100 μl. The tumor-initiating capacity of the two populations was monitored weekly and compared. Animals were euthanized when tumors exceeded 1, 800 mm^3^ or the 60-day endpoint was reached.

### Western blot assay for P-gp in sorted cells

Both ALDH^br^ and ALDH^lo^ subpopulations isolated from 4T1 cells were washed with phosphate-buffered saline (PBS). The cells were collected by centrifugation, and the pellet was suspended in RIPA lysis buffer (Biomiga, Inc.) containing a cocktail of proteinase inhibitors i.e., leupeptin, aprotinin and pepstatin (the first two agents were purchased from Roche, and pepstatin was purchased from Amresco). The cells were lysed on ice for 15 minutes. The lysates were centrifuged at 12, 000 rpm for 10 minutes at 4°C, and the protein in the supernatant was collected. The protein concentration was quantified using the bicinchoninic acid (BCA) assay kit (Applygen Technologies, Inc.) to ensure that equal amounts of protein from both subpopulations were loaded in the gel. The remaining supernatants were boiled in sample buffer containing sodium dodecyl sulfate (SDS) and β-mercaptoethanol and then used for western blot analysis.

Western blot analysis was performed as previously described with slight modifications [38]. Briefly, the proteins were separated using a 7% sodium dodecyl sulfate–polyacrylamide gel electrophoresis (SDS-PAGE). Following electrophoresis, the samples were transferred onto a polyvinylidene difluoride (PVDF) membrane. After the transfer, non-specific binding sites were blocked at room temperature for 1 hour with 5% non-fat dry milk in PBS with gentle agitation. The membranes were incubated overnight at 4°C with the following primary antibodies: monoclonal mouse anti-P-gp (JSB-1, Abcam) (1:200 dilution in 3% non-fat dry milk) and anti-β-actin (1:1, 000 dilution, Zhongshan goldenbridge biotechnology Co., Ltd.), which was used for normalization. The membrane was washed with PBS (3 × 10 minutes) and then incubated with a horseradish peroxidase-conjugated goat anti-mouse IgG (1:5, 000 dilution, Zhongshan goldenbridge biotechnology Co., Ltd.) for 1 hour at room temperature with gentle agitation followed by rinsing as before. The protein bands were visualized using an enhanced chemiluminescence (ECL) detection system (Applygen Technologies Inc.), according to the manufacturer’s instructions, followed by exposure to X-ray film.

### *In vitro* cytotoxicity of oncolytic HSV1

For 4T1 monolayers, the day before infection, 2 × 10^5^ 4T1 cells were seeded into 6-well plates. After a 24-hour incubation, the cells of one well were trypsinized and counted to facilitate the calculation of the MOIs for infection. The media in the other wells were replaced with 1 ml fresh DMEM/F12 SFM containing viruses at different MOIs. One control well was added only DMEM/F12 SFM. After a 1-hour incubation, 1 ml of DMEM/F12 full growth medium (FGM) was added to each well. The CPE was observed at different times after infection using an inverted phase contrast microscope.

For the mammospheres, after 7 days of culture, 4T1 mammospheres of one well were collected and digested with Accutase® solution (Sigma), and the cells were counted to calculate the MOIs for virus infection. The mammospheres of the other wells were infected with HSV1-GFP at an MOI of 1, and a well with the addition of vehicle was used as a control. HSV1-GFP was directly added without changing the medium. The GFP expression was observed at different time points.

For ALDH^br^ and ALDH^lo^ cells, the cells were resuspended in DMEM/F12 FGM and seeded into dishes (33 mm in diameter) at a density of 5 × 10^4^ cells/ml in 2 ml/dish. The media was then replaced with 1 ml of fresh DMEM/F12 SFM containing HSV1-GFP at different MOIs when most cells were adherent. One control dish contained DMEM/F12 SFM only. After a 1-hour incubation, the medium was changed with 2 ml of fresh DMEM/F12 FGM, which contained just 3% FCS to keep the cells alive but to slow cell differentiation. After incubation for another 10 hours, GFP expression was examined, and images were obtained using an inverted fluorescence microscope.

### Establishment of a subcutaneous cancer model and tumor therapy protocols

5–6 weeks old immune-competent female Balb/c mice were obtained from the Institute of Zoology, Chinese Academy of Sciences. All protocols for the animal experiments were approved by the Animal Care and Use Committee of the Cancer Institute and Hospital, Chinese Academy of Medical Sciences. All mice were housed and handled according to the institutionally recommended guidelines. A total of 5 × 10^4^ 4T1 cells were s.c. injected into the right flank of mice. After 4–5 days, when tumors appeared, the mice were distributed by tumor size into the following treatment groups (n=12-13 per group): (a) doxorubicin followed by HSV1, (b) doxorubicin alone, (c) HSV1 alone and (d) control. Chemotherapy with 8 mg/kg doxorubicin was i.v. administered twice at days 0 and 3. Oncolytic HSV1-hGM-CSF treatment at a dose of 1 × 10^7^ plaque forming units (PFU) per mouse was subsequently given by direct intratumoral injection on days 5, 7, 9, 11 and 13. We treated the control mice with the solvent for doxorubicin and oncolytic HSV1 (NS and DMEM/F12 SFM, respectively). The primary tumor size and body weight were routinely measured every 4 days following treatment. The tumor volume was calculated using the following formula: tumor volume (mm^3^) = [L × W^2^] / 2, where L equals length and W equals width in mm.

After the third treatment with oncolytic HSV1-hGM-CSF (on day 11), the primary tumors were surgically removed from each group (n=4-5) and used for the aldefluor assay. The remaining animals in each group were used for the survival analysis (n=5-7).

### Flow cytometry analysis of 4T1 cells with ALDH^br^ activity

4T1 cells at a density of 1 × 10^5^ cells/ml were seeded in T-75 cm^2^ cell culture flasks at 15 ml/flask. On the second day, the medium was changed with fresh DMEM/F12 FGM, and the cells were cultured for an additional 24 hours in the presence or absence of doxorubicin (1.0 μg/ml) or HSV1-hGM-CSF (MOI=0.3). The controls were incubated with vehicle only. Then the cells were harvested, and a single-cell suspension was obtained for the aldefluor assay as described above.

Primary tumor tissue comprising 4T1 cells was isolated, minced into tiny fragments and digested with collagenase IV (1 mg/ml) and DNase I (300 U/ml) (all obtained from Sigma) for approximately 1.5 hours at 37°C in 5% CO_2_ with intermittent pipetting. The single cells obtained by filtering through a 200-mesh screen were lysed with Ammonium Chloride Solution (Stem Cell Technologies) to exclude red blood cells. After the aldefluor assay was performed, the cells were stained with APC anti-mouse CD45 (BioLegend) and its isotype antibody at 4°C for 30 minutes to exclude leukocytes. The ALDH^br^ cells were then analyzed by flow cytometry as described above.

### Characterization of CD8^+^ T lymphocytes in splenocytes by flow cytometry analysis

After the second treatment with oncolytic HSV1 (on day 8), the spleens (n=3-4) were surgically removed and used for CD8^+^ T lymphocyte measurement, and their cytotoxicity to 4T1 ALDH^br^ cells was measured by flow cytometry. The single cell suspension was prepared through a 400-gauge mesh. Lymphocytes from the spleens were isolated by centrifugation in gradient lymphocyte isolation solution for mice (Tianjin Hao Yang Biological Manufacture Co., Ltd., China) at room temperature and washed twice with PBS. The cell suspensions in PBS were then stained at 4°C for 30 minutes using the following antibodies: FITC anti-mouse CD3, PE anti-mouse CD8b and their corresponding isotype control antibodies (all monoclonal antibodies were obtained from Biolegend). After washing with PBS, the cells were fixed with 10% formaldehyde and the CD8^+^ T lymphocyte frequency was determined by flow cytometry.

### CTL cytotoxicity assay by flow cytometry

*In vitro* CTL detection was performed as previously described with some modification [39]. ALDH^br^ cells were sorted from 4T1 cells on the day of the assay. To label target cells, the isolated cells were resuspended in PBS containing 5-(and −6) -carboxyfluorescein diacetate succinimidyl ester (CFSE, Sigma) at a final concentration of 10 μmol/l and incubated at 37°C/5% CO_2_ for 10 minutes. After incubation, the reaction was stopped by adding a large volume of DMEM/F12 FGM, and the labeled target cells were then washed in DMEM/F12 FGM to sequester any free CFSE. After resuspension in DMEM/F12 FGM, the cell number and viability were assessed using trypan blue exclusion, and the cell concentration was adjusted to 2 × 10^4^ viable cells/100 μl with DMEM/F12 FGM. Effector cells (lymphocytes) were obtained from euthanized tumor-bearing mice from the different treatment groups as described above. Isolated lymphocytes were washed and resuspended in DMEM/F12 FGM, the cell number and viability were assessed as described above, and the cell concentration was adjusted to 2 × 10^6^ viable cells/100 μl. Effector and target cells were mixed at an E:T ratio of 100:1 (2 × 10^6^ for effector cells and 2 × 10^4^ for target cells, respectively) in a total volume of 200 μl per test. After incubation for 3.5 hours at 37°C/5% CO_2_, DMEM/F12 FGM was added to a final volume of 0.5 ml and stained with propidium iodide (PI, Sigma) at a final concentration of 2 μg/ml. Finally, cytotoxicity against the target cells was assessed by flow cytometry. The determination of cytotoxicity was based on enumeration of dead target cells (CFSE^+^PI^+^). To determine specific lysis, the survival of allogeneic splenocytes in tumor-free naive mice was used as a baseline. All of the assays were performed in triplicate.

### Statistical analysis

All statistical tests were performed using GraphPad Prism 5 software. The Student's unpaired t-test was performed on the following experimental data: the frequency of ALDH^br^ cells and CD8^+^ T lymphocytes and tumor volume. The results are presented as the mean ± SEM (standard error of the mean). The Kaplan–Meier method and the log-rank test were used to compare survival, which was defined as the time from tumor inoculation until the endpoint. All statistical tests were two-sided, and statistical significance was defined as a p < 0.05.

## Results

The scheme for construction of the two recombinant oncolytic viruses, HSV1-GFP and HSV1-hGM-CSF, is shown in Figure 1. They are attenuated by the deletion of both copies of the ICP34.5, which is neurovirulence gene, and of ICP 47 gene. Deletion of gene encoding ICP34.5 provides tumor selectivity. ICP47 functions to block antigen processing in HSV1 infected cells, and therefore deletion of it is aiming to improve anti-tumor immunity [40]. Deletion of ICP47 also results in the US11 gene being under the control of the ICP47 immediate early promoter that results in enhanced virus replication. Insertion of GFP gene or hGM-CSF is to serve as an infection marker or to improve vector’s immune stimulating potency, respectively.

### 4T1 ALDH^br^ cells have CSC characteristics

**Figure 2:**
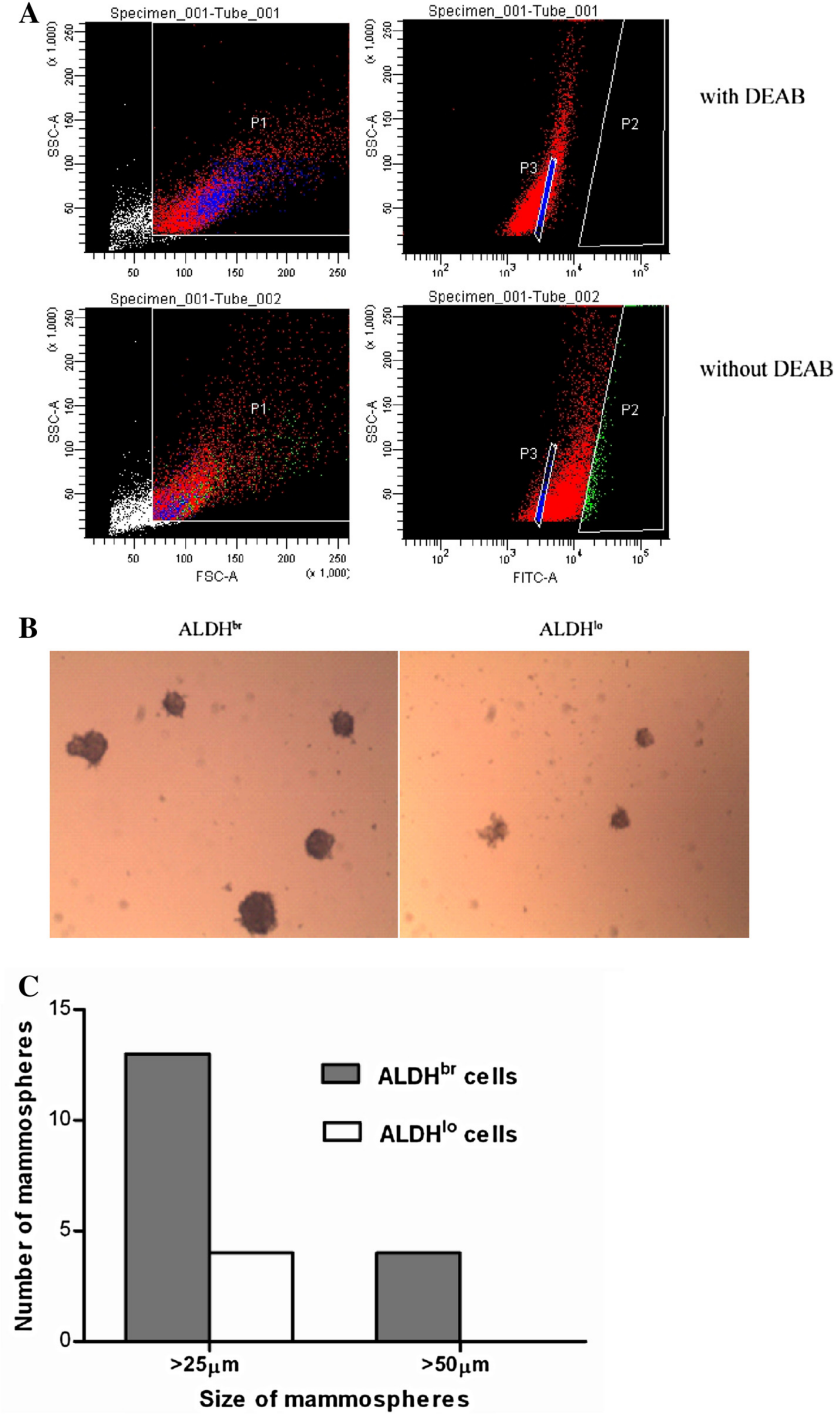
**4T1 ALDH**^**br **^**cells have high mammosphere-forming ability. **(**A**) Representative flow cytometry plots demonstrating the sorting of 4T1 cells with high (ALDH^br^, P2, 3~5% of P1 gate) and low ALDH activity (ALDH^lo^, P3, 3~5% of P1 gate). The first gate (P1) chooses the cells with good status from the total cells and excludes cell debris according to their FSC & SSC values. The cells were incubated with an aldefluor substrate (BAAA), and the specific inhibitor of ALDH, DEAB, was first used to establish the baseline fluorescence level of ALDH activity (top). Staining of 4T1 cells with aldefluor substrate but without DEAB inhibitor produced a shift in BAAA fluorescence that defined the ALDH^br^ population (bottom). (**B**) ALDH^br^ (left) and ALDH^lo^ (right) cells were plated for mammosphere formation (5 × 10^4^ cells/well) for 8 days. Mammospheres were observed using an Olympus inverted phase contrast microscope (40 × objective magnifications). (**C**) The data shown are the average number of spheres counted in different sizes from a representative experiment performed in duplicate wells.

In some tumor cells, including 4T1 cells, the ability to form spherical aggregates (“spheres”) in non-adherent culture conditions has been shown to be a characteristic of CSCs [41-43]. In this study, CSCs were identified and isolated from 4T1 cells using the aldefluor assay. To determine whether these sorted 4T1 cells with high ALDH1 enzymatic activity, termed “ALDH^br^” cells, are bona fide CSCs, two aspects were examined: the mammosphere-forming ability *in vitro* and tumorigenicity *in vivo*. ALDH^br^ and ALDH^lo^ 4T1 subpopulations were isolated by flow cytometry (Figure 2A). As shown in Figures 2B and C, ALDH^br^ cells gave rise to approximately 3-fold more tumorspheres than the ALDH^lo^ cells when the mammospheres were larger than 25 μm in size. In addition, mammospheres larger than 50 μm were only produced by ALDH^br^ cells.

The tumorigenic ability *in vivo* is the gold standard for identifying CSCs, and it has been studied in many tumors [44,45]. To investigate the possible difference in tumor formation potential between the ALDH^br^ and ALDH^lo^ subpopulations, serial dilutions of both subpopulations were s.c. injected into Balb/c mice (as described above). Both subpopulations were able to form tumors when 5, 000 or more cells were implanted (Table 2). However, when 1, 000 or fewer cells were implanted, the frequency of tumor formation was higher for ALDH^br^ than for ALDH^lo^ cells. At the 1, 000-cell dilution, the ALDH^br^ cells formed tumors in 5/5 mice, whereas the ALDH^lo^ cells formed tumors in 2/5 mice. Remarkably, as few as 100 ALDH^br^ cells were able to form tumors in 4/6 mice, whereas the same number of ALDH^lo^ cells failed to generate tumors in any mice (0/6) over 2 months.

**Table 2:** Tumor-initiating capacity of freshly sorted ALDH^**br **^*versus *ALDH^**lo **^subpopulations *in vivo*

**Subpopulation**	**ALDH**^**br**^			**ALDH**^**lo**^		
Cell numbers	5, 000	1, 000	100	5, 000	1, 000	100
Tumor incidence	5/5	5/5	4/6	5/5	2/5	0/6

Serial dilutions of ALDH^br^*versus* ALDH^lo^ cells were s.c. injected into female Balb/c mice, and tumor growth was observed weekly and compared. The tumor formation frequency between both subpopulations was plotted against the number of cells injected (i.e., 5, 000, 1, 000 and 100 cells).

Collectively, the 4T1 ALDH^br^ cells had increased mammosphere-forming capacity and tumor-initiating potency compared with ALDH^lo^ cells; thus, they possessed CSC properties.

### 4T1 ALDH^br^ cells are resistant to chemotherapy

**Figure 3:**
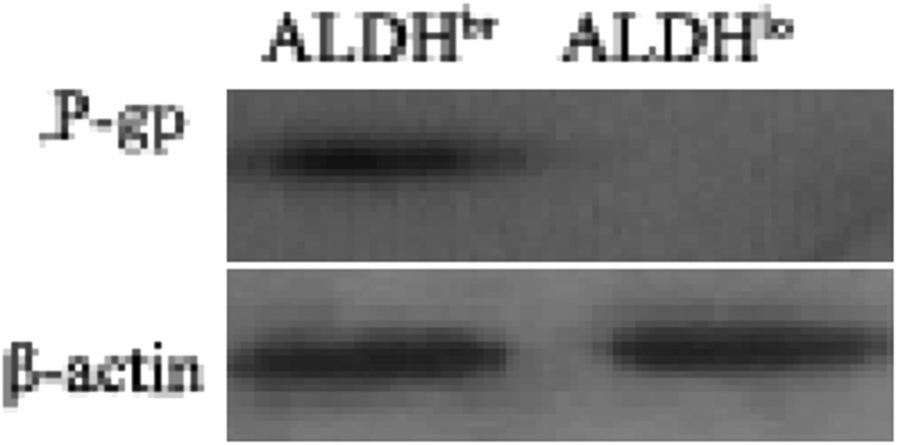
**Western blot analysis demonstrated increased P-gp expression in 4T1 ALDH**^**br **^**cells. **The western blot analysis was performed as described in the Methods section. P-gp (141 kDa) expression was significantly increased in 4T1 ALDH^br^ cells compared with ALDH^lo^ cells. β-actin served as the loading control. The experiment was performed in duplicate with similar findings obtained in each experiment. A representative blot is shown.

CSCs are responsible for resistance to conventional chemotherapy. P-gp is considered to play an important role in the development of chemoresistance in breast cancer [46]. Western blot analysis was performed to determine the expression of P-gp in the sorted ALDH^br^ and ALDH^lo^ subpopulations of 4T1 cells. The expression of P-gp was significantly elevated in ALDH^br^ cells when compared with the corresponding ALDH^lo^ cells in which P-gp expression was not detectable, whereas there was no significant change in the expression of β-actin in both subpopulations (Figure 3). The different expression levels of P-gp might well explain why 4T1 ALDH^br^ cells were chemoresistant.

### Both 4T1 monolayers and mammospheres are infectable to HSV1 *in vitro*

**Figure 4:**
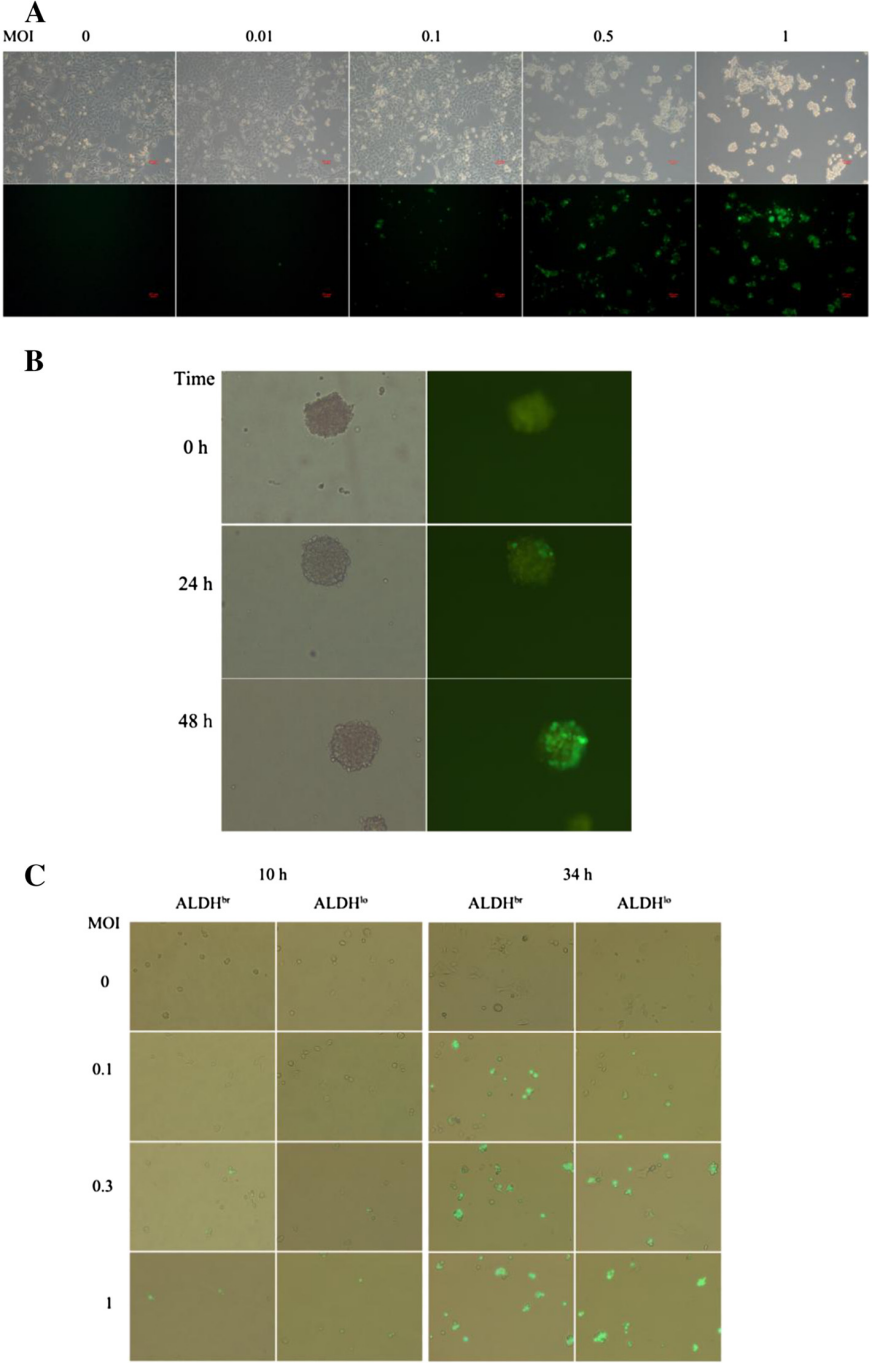
**HSV1-GFP was highly oncolytic to 4T1 monolayers, mammospheres and isolated ALDH**^**br **^**and ALDH**^**lo **^**cells. **(**A**) 4T1 cells were infected with HSV1-GFP at different MOIs (0, 0.01, 0.1, 0.5 and 1) for 24 hours. The images were visualized using a phase-contrast microscope (top) and a fluorescence microscope for GFP expression (bottom). (**B**) After a 7 day culture, 4T1 mammospheres were infected with HSV1-GFP at an MOI of 1. At 24 (middle) and 48 (lower) hours after infection, the spheres exhibited a GFP signal compared with the mock-infected control (upper). The images were visualized using phase-contrast (left) and fluorescence microscopy (right). (**C**) Sorted ALDH^br^ and ALDH^lo^ cells were infected by HSV1-GFP at different MOIs (0, 0.1, 0.3 and 1). The cell morphology and GFP expression were visualized using an inverted fluorescence microscope, and overlapping images were taken at 10 (left panels) and 33 hours (right panels) after infection (100 × objective magnifications).

To investigate the infection of 4T1 mammospheres by oncolytic HSV1, HSV1-GFP was used to easily observe infected mammospheres with green fluorescence. First, the cell-killing ability of HSV1-GFP against 4T1 tumor cell monolayers was investigated *in vitro*. By 24 hours after infection, the infected monolayer cells displayed a marked CPE (nearly 85% at an MOI of 0.5, 95% at an MOI of 1) compared with control mock-infected cells (Figure 4A). Typical CPE cells can be characterized by rounded and contracted shapes, detachment from one another, or even detachment from the tissue culture plate.

Mammosphere cultures from the 4T1 cell line were established and infected by oncolytic HSV1-GFP. Strong green fluorescence due to constitutive GFP expression was detected in many cells of the spheres compared with the mock-infected control (Figure 4B). At an MOI of 1 for 24 hours, HSV1-GFP could infect a few cells of mammospheres, while at a later time of infection (48 hours), it had spread into more cells of the mammospheres. Collectively, these data indicate that both 4T1 monolayers and mammospheres can be infected by oncolytic HSV1-GFP.

### ALDH^br^ and ALDH^lo^ cells can be uniformly infected by HSV1-GFP

4T1 cells stained with aldefluor substrate demonstrated bright green fluorescence, which could not be distinguished from the GFP expression in cells infected with HSV1-GFP. However, the green fluorescence quickly disappeared from post-sorted ALDH^br^ cells because they were no longer in the aldefluor buffer, which blocked ABC transporters and retained the fluorescent substrate in the cells, but were instead in sheath fluid and medium when plated in dishes. Therefore, the isolated ALDH^br^ cells were fluorescent-free and HSV1 drived GFP expression could be used to evaluate the HSV1 infectivity to ALDH^br^ cells. Sorted ALDH^br^ and ALDH^lo^ cells were infected with HSV1-GFP at different MOIs for different times. As shown in Figure 4C, after 10 hours, GFP expression had appeared in both subpopulations infected with HSV1-GFP at MOIs of 0.3 and 1. After infection for 34 hours, when cell proliferation and differentiation had occurred, GFP expression was more apparent in both subpopulations. Moreover, the HSV1-GFP infection efficiency in both ALDH^br^ and ALDH^lo^ cells was similar (38.8% for ALDH^br^ cells versus 29.4% for ALDH^lo^ cells, respectively, p=0.0664, n=4-6).

### 4T1 ALDH^br^ cells are resistant to doxorubicin but treatable to HSV1 *in vitro* and *in vivo*

**Figure 5:**
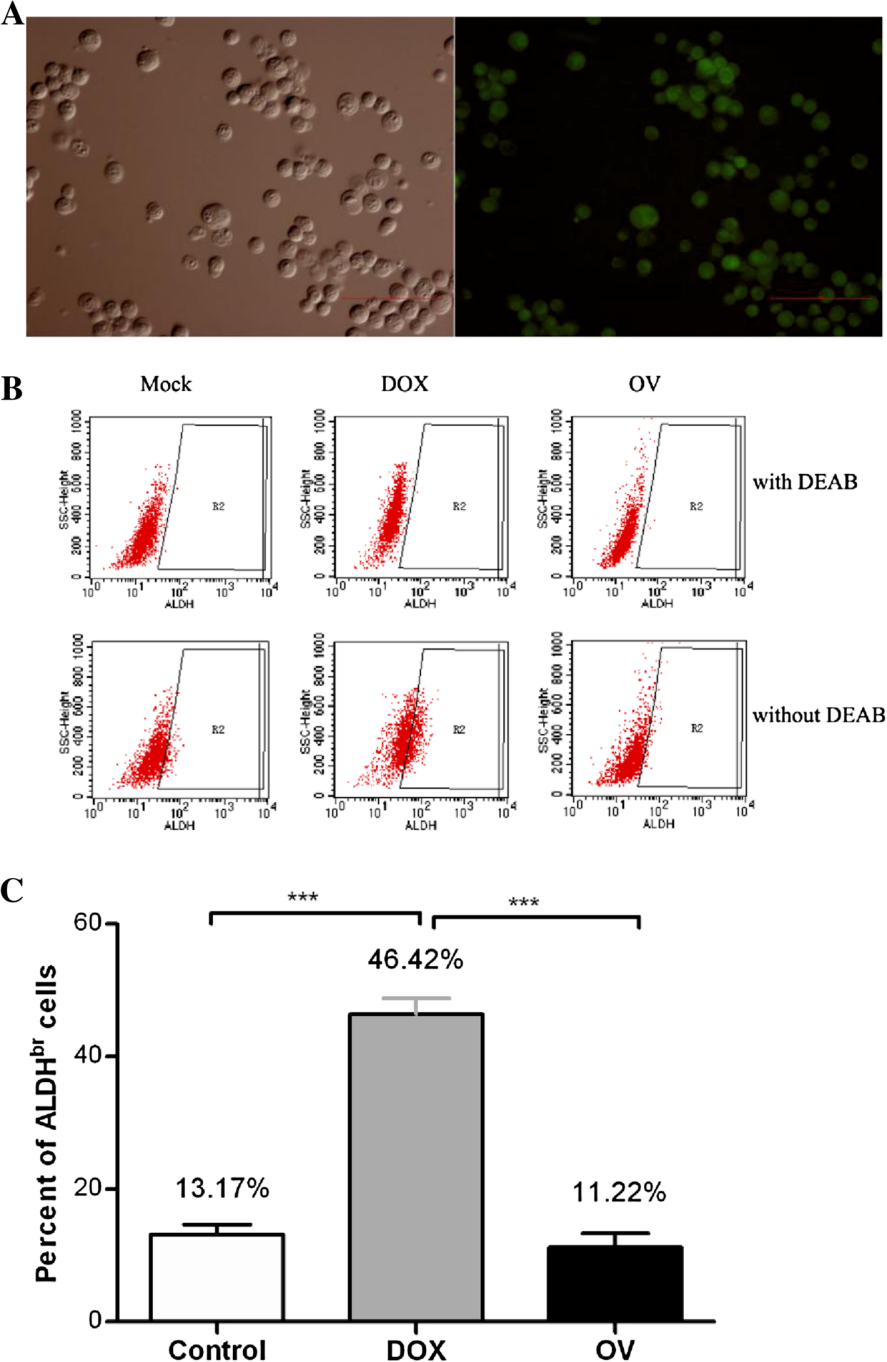
**The effect of different *in vitro *treatments on 4T1 ALDH**^**br **^**cells was assessed using flow cytometry. **(**A**) 4T1 cells stained with the aldefluor substrate were imaged using phase-contrast (left) and fluorescence microscopy (right) (100 × objective magnifications). (**B**) An aldefluor assay of ALDH^br^ in 4T1 cells treated with either doxorubicin (1.0 μg/ml, middle), HSV1-hGM-CSF (MOI=0.3, right), or media alone (Mock) (left). The upper images in each column are the DEAB-treated negative control, and the lower pictures are the corresponding experimental sample. (**C**) The ALDH^br^ cell frequency in the presence of doxorubicin or HSV1-hGM-CSF was compared with the control. The data represent the mean ± SEM of three independent experiments. ***, p<0.001 for a significant difference between the doxorubicin-treated group and the other two groups. Abbreviations: DOX, doxorubicin; OV, HSV1-hGM-CSF.

To further examine the effect of oncolytic HSV1 on 4T1 ALDH^br^ cells, 4T1 cells were treated with doxorubicin (1.0 μg/ml) or HSV1-hGM-CSF (MOI=0.3) for 24 hours (when a similar CPE, approximately 30%, appeared in both treated cells, data not shown) and subsequently investigated for changes in the proportion of ALDH^br^ cells by flow cytometry. Because of the overlapping fluorescence between the activated aldefluor substrate (Figure 5A) and GFP expression by flow cytometry, oncolytic HSV1-hGM-CSF was used instead. There was a 3.5-fold increase in the proportion of ALDH^br^ cells after treatment with doxorubicin (up to 46.42%) *versus* the corresponding control (13.17%) (n=3, p<0.0001, Figures 5B and C). However, the presence of HSV1-hGM-CSF in the medium did not significantly alter the percentage of ALDH^br^ cells in the 4T1 cells (11.22%) compared with the control (n=3, p=0.47). The results demonstrated that 4T1 ALDH^br^ cells, which could be significantly enriched by doxorubicin *in vitro,* could be uniformly killed by oncolytic HSV1.

**Figure 6:**
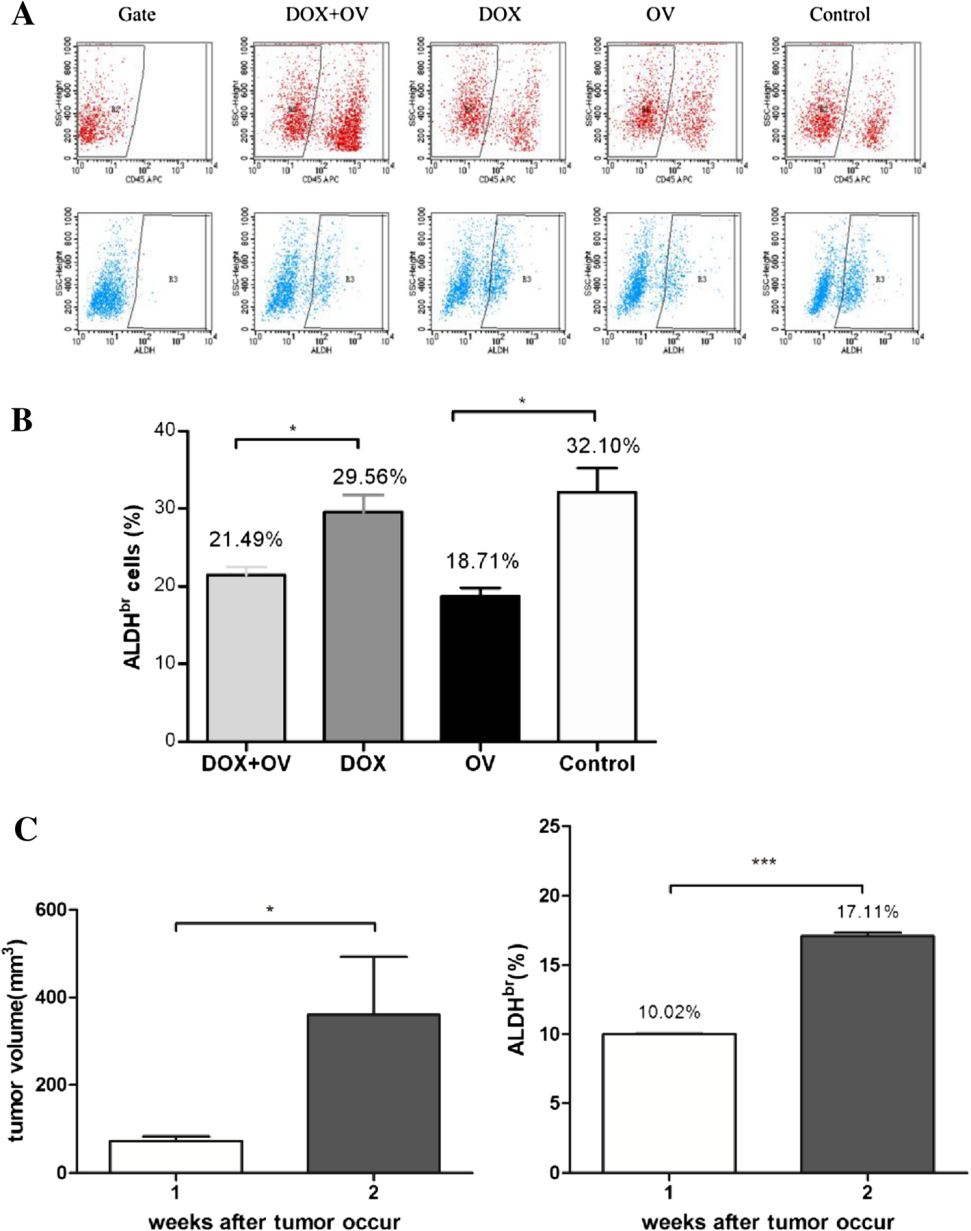
**The influence of doxorubicin and/or HSV1-hGM-CSF on ALDH**^**br **^**tumor cells *in vivo*. **(**A**) An aldefluor assay of ALDH^br^ in *in vivo*-treated primary tumors as described in the Methods section. Representative images of flow cytometry from the four groups are depicted. The upper panels are the gates to exclude APC anti-mouse CD45^+^ leukocytes. The percent of CD45^-^ cells was as followed: 33.45% for DOX+OV, 58.89% for DOX, 56.54% for OV and 61.69% for Control. (**B**) The mean frequency of ALDH^br^ tumor cells in the four groups are as follows (n=4-5). The experiment was performed twice with similar findings. (**C**) The influence of the primary tumor volume on 4T1 ALDH^br^ cells *in vivo*. The mean tumor volume (left), and the mean percentage of ALDH^br^ tumor cells (right, n=3-5) both increased with time. The data represent the mean ± SEM in triplicate. *, p < 0.05; ***, p < 0.001. Abbreviations: DOX, doxorubicin; OV, HSV1-hGM-CSF.

To determine whether these ALDH^br^ tumor cells also exhibited resistance to doxorubicin chemotherapy or sensitivity to HSV1-hGM-CSF *in vivo*, the ALDH^br^ tumor cell frequency was examined *in vivo* after different treatments. Inconsistent with the *in vitro* data, there was no further increase in the ALDH^br^ tumor cell frequency *in vivo* after the chemotherapy-alone treatment, which was similar to, or even slightly less than, those of the tumors treated with vehicle (29.56% for doxorubicin *versus* 32.10% for vehicle, n=3, p>0.05) (Figures 6A and B). In contrast, oncolytic HSV1-hGM-CSF single-agent therapy resulted in a significant decrease in the ALDH^br^ tumor cell content (18.71%, n=3, p<0.05 for HSV1 *versus* control), and the frequency of the ALDH^br^ tumor cells was also dramatically reduced in tumors treated with doxorubicin followed by HSV1-hGM-CSF (21.49%) *versus* vehicle or doxorubicin alone (n=3, p<0.05).

### ALDH^br^ subpopulation increased with the development of a primary tumor *in vivo*

We speculated that the reason the percentage of ALDH^br^ 4T1 cells in the control group was unexpectedly high (32.10%, Figure 6B) may be because of their larger tumor volume. Therefore, 4T1 tumor-bearing mice with different primary tumor volumes (1 week and 2 weeks after the tumor occurred) were selected, and the change in the percentage of ALDH^br^ 4T1 cells in the primary tumors was analyzed. As expected, the frequency of ALDH^br^ tumor cells grew with the development of primary tumors (Figure 6C). The mean primary tumor volume was 73.0 mm^3^ at 1 week and 360.5 mm^3^ at 2 weeks after the tumor appeared (n=3, p=0.0437), and the frequency of ALDH^br^ tumor cells was 10.02% and 17.11%, respectively (n=3, p<0.0001). Therefore, the percentage of ALDH^br^ 4T1 cells increased with the growth of primary tumors.

### CD8^+^ T lymphocytes induced by HSV1 does not appear to be responsible for ALDH^br^ tumor cell eradication

To explore whether the eradication of ALDH^br^ tumor cells was mediated by CD8^+^ T lymphocytes generated in mice after oncolytic HSV1 treatment, we assessed the CD8^+^ T lymphocyte percentage and activity after different treatments.

**Figure 7:**
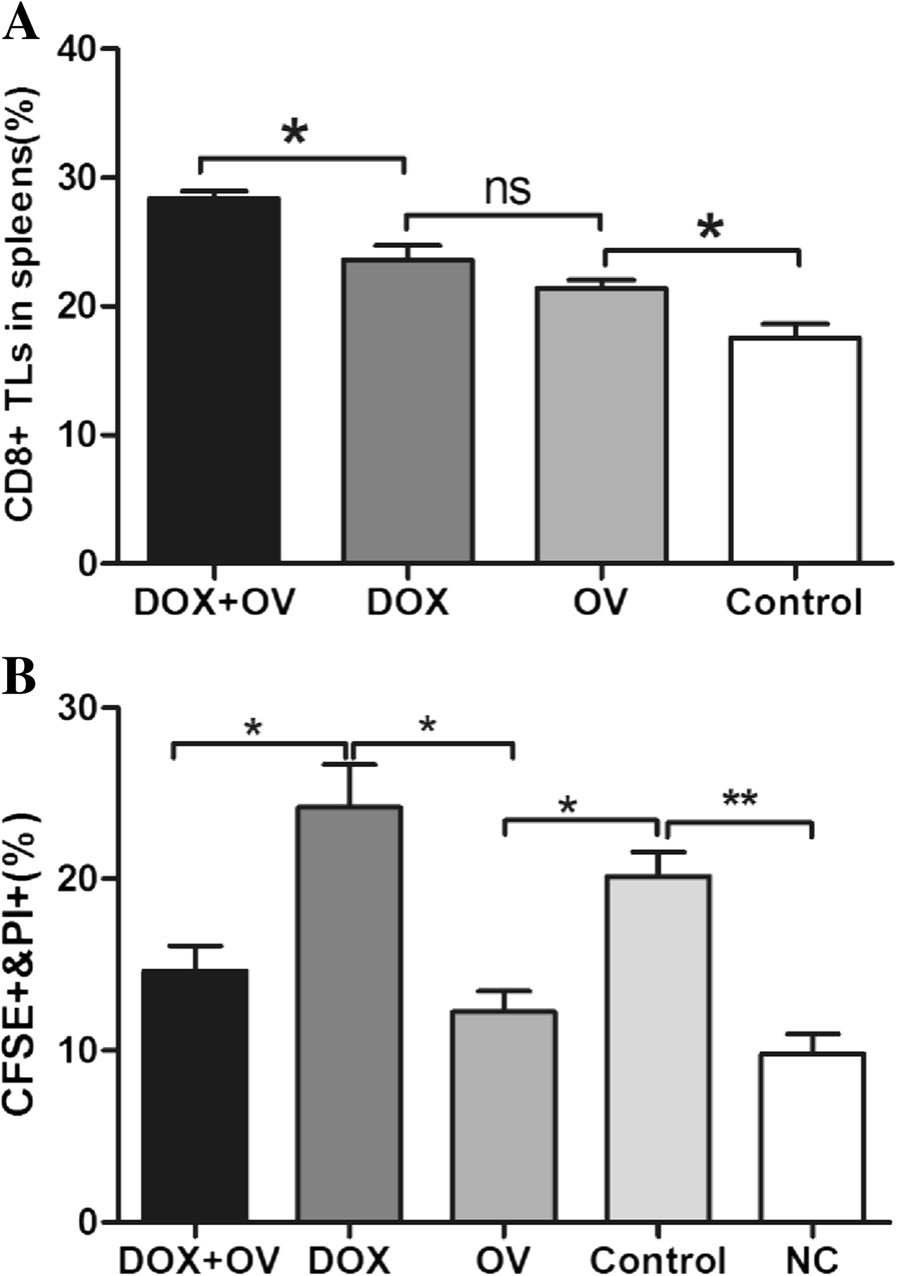
**Cytotoxic activity to 4T1 ALDH^**br **^of CD8^**+ **^T lymphocytes from mice following different therapies. **(**A**) The proportion of CD8^+^ T lymphocytes in spleens after treatments. (**B**) CD8^+^ T lymphocyte cytotoxicity to 4T1 ALDH^br^ by CFSE/PI flow cytometry. Negative control splenocytes were taken from tumor-free naive mice. Statistical analysis was performed using the unpaired Student’s t test: *, p < 0.05; **, p < 0.01; ns, not significant. Abbreviations: DOX, doxorubicin; OV, oncolytic HSV1-hGM-CSF; TLs, T lymphocytes; NC, negative control.

Flow cytometric analysis of the frequency of CD8^+^ T lymphocytes in spleens from mice with different treatments and their results were followed. As shown in Figure 7A, treatment with either doxorubicin or oncolytic HSV1 had a marked effect on the CD8^+^ T lymphocyte frequency in the spleens (23.63% and 21.40%, respectively) compared with the control group (17.53%) (n=3, p<0.05). The CD8^+^ T lymphocyte frequency in spleens treated with doxorubicin demonstrated a slight elevation compared with that of the oncolytic HSV1 group, but no significant difference existed (n=3, p>0.05). The proportion of CD8^+^ T lymphocytes demonstrated a greater increase in mice treated with doxorubicin followed by oncolytic HSV1 (28.37%; n=3; p<0.05).

CD8^+^ T lymphocytes induced by doxorubicin demonstrated significant cytotoxic activity to 4T1 ALDH^br^ cells with an E/T ratio of 100:1 (24.17%) (n=3, p<0.05), while lymphocytes from mice treated with oncolytic HSV1 did not seem to demonstrate significant CTL activity against the same target cells at the same E/T ratio (12.27%), which was even less than that of the control group (20.13%) (n=3, p<0.05) (Figure 7B). Moreover, cytotoxic activity induced by doxorubicin plus oncolytic HSV1 treatment increased slightly compared with the nonspecific cytotoxicity (14.67% versus 9.80%, respectively; n=3; p>0.05), which was also lower than that of the control group (n=3, p>0.05).

### Doxorubicin followed by HSV1 demonstrated the greatest therapeutic effect *in vivo*

**Figure 8:**
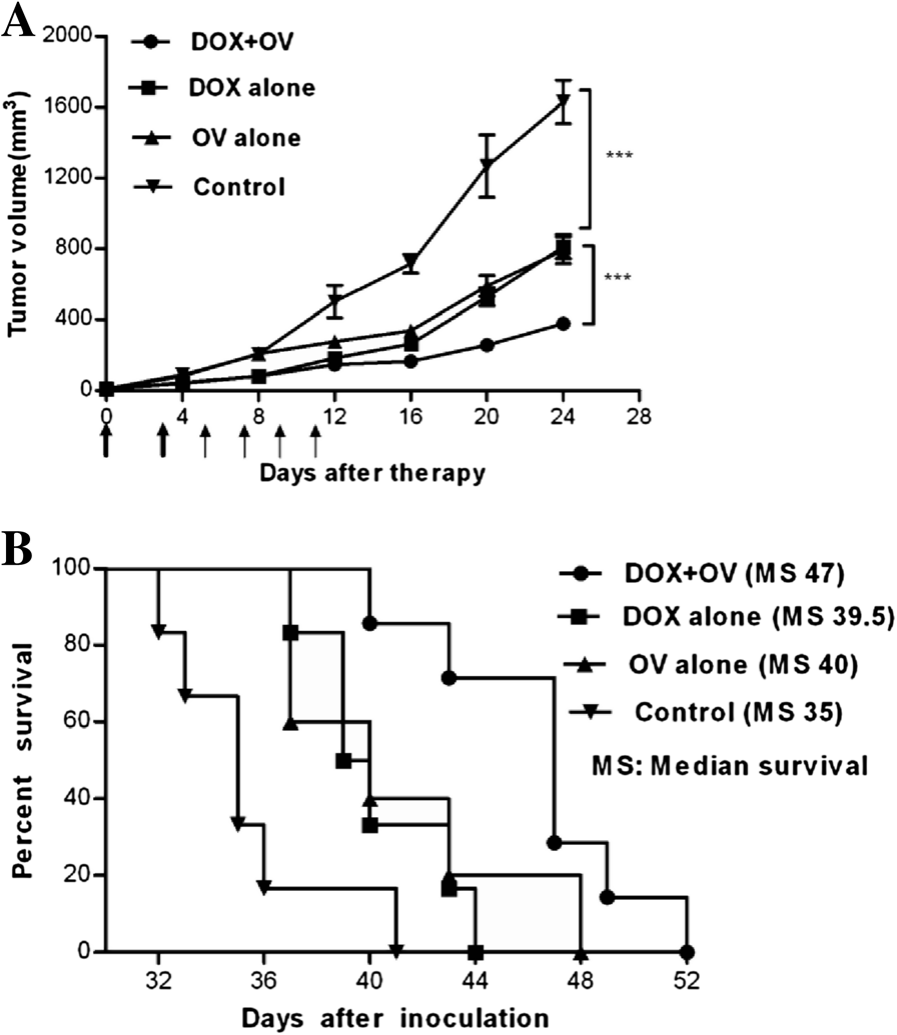
**Anticancer effect of doxorubicin and/or HSV1-hGM-CSF against 4T1 breast tumors. **The mice bearing 4T1 tumors were treated with doxorubicin and/or HSV1-hGM-CSF as described in the Methods section. (**A**) The tumor volume of mice among the different groups was measured every 4 days following treatments. The data represent the mean ± SEM (n=6). Arrows indicate the type of treatments: thick arrows for doxorubicin chemotherapy and thin arrows for oncolytic HSV1-hGM-CSF treatment. (**B**) The median survival times for the 4 groups are illustrated in Kaplan–Meier survival curves (n=5-7). ***, p < 0.001. The experiment was performed twice with similar findings. Abbreviations: DOX, doxorubicin; OV, HSV1-hGM-CSF.

The above observations provided a rationale for further evaluating the treatment modalities with respect to their overall anticancer effects *in vivo*. 4T1 breast tumors treated with either doxorubicin or oncolytic HSV1-hGM-CSF experienced a significant reduction in tumor volume compared with the vehicle-treated control group (n=6, p<0.001). Moreover, the combined treatment resulted in a more significant reduction in tumor volume compared with the other two treatment groups (n=6, p≤0.0001) (Figure 8A). No statistical significance was observed between the doxorubicin-alone and HSV1-hGM-CSF-alone treatment groups.

None of the animal tumors completely regressed, and all animals died due to excessive tumor growth. Mice treated with doxorubicin or HSV1-hGM-CSF alone demonstrated a prolonged median survival time compared with mock-treated mice (35 days for the control group, 39.5 days for the doxorubicin-alone group and 40 days for the HSV1-alone group; n=5-7; p<0.05, log-rank test) (Figure 8B). The median survival time between mice that received either treatment alone was not significantly different. For the combination group, the median survival time was significantly longer (47 days) compared with either treatment alone (n=5-7, p<0.01 *versus* doxorubicin-alone, p>0.05 *versus* HSV1-alone, log-rank test). The results above demonstrated that treatment with either doxorubicin or HSV1 exhibited significant anticancer effects, and the combination treatment group demonstrated the greatest anticancer efficacy *in vivo*.

## Discussion

CSC chemoresistance has become the main obstacle in successful anticancer treatment and is largely responsible for human breast cancer mortality [47-49]. Cells with high ALDH1 activity have been shown to possess CSC characteristics in many tumor types [13-18]. In our study, ALDH^br^ cells isolated from 4T1 cells were shown to have CSC properties, including their mammosphere-forming ability *in vitro* and tumorigenicity *in vivo*. In addition, CSCs typically overexpress cell membrane ATP-binding cassette (ABC) drug transporters such as P-gp, which mediate the efflux of a large number of cytotoxic compounds, including doxorubicin. Our study indicated that P-gp expression was only detected in 4T1 ALDH^br^ cells.

Among the therapeutic agents used for human breast cancer, doxorubicin, an anthracycline drug, remains a first-line choice [50,51]. Despite its excellent anticancer activity in the clinic, doxorubicin treatment is confronted with toxicities, including severe myelosuppression and dose-cumulative cardio-toxicity [52-54]. Most importantly, similar to many other chemotherapeutic drugs, the treatment of breast cancer with doxorubicin was unable to eradicate CSCs but instead led to an enrichment of CSCs [55,56]. HSV1 is a potent oncolytic virus that has been evaluated in many types of tumors in mice and humans [27-29,57]. We compared the effects of oncolytic HSV1 and doxorubicin on the ALDH^br^ population *in vitro* and *in vivo*. Our *in vitro* results, in agreement with previous reports [55,56,58], indicated that doxorubicin can enrich the CSCs of 4T1 cells, which were still treatable by oncolytic HSV1. Phenotypic analysis revealed an obvious decrease in the frequency of ALDH^br^ tumor cells in mice treated with oncolytic HSV1. It has been reported that oncolytic HSV can induce a robust anti-tumor immune responses against 4T1 mammary tumors *in vivo*[27,59,60] and CTLs mediated cytotoxicity to cancer cells [35]. In our study, though the CD8^+^ T lymphocyte frequency in spleens treated with oncolytic HSV1 demonstrated a significant elevation compared with the control group, no significant specific CTL response appeared to participate in ALDH^br^ cell elimination. This may be due to the following reasons: (1) The CD8^+^ T lymphocytes induced by oncolytic HSV1 may be mainly specific to the virus itself (a strong immunogen compared with 4T1 cells) and this was supported by marked splenomegaly observed after oncolytic virus alone treatment (data not shown), and (2) the hGM-CSF carried by oncolytic HSV1 was ineffective in mice due to species differences [61]. Unexpectedly, there was no increase in the percentage of ALDH^br^ cells in tumors treated with doxorubicin alone compared with those treated with vehicle. This unexpected result may be due to the following factors: (1) The time between the last chemotherapy and aldefluor assay was too long (8 days) to exam the enrichment of CSCs from doxorubicin, (2) Doxorubicin by itself can enhance the anticancer immune response [62,63] and may also enhance the anti-CSC activity (Figure 7B), which may explain why ALDH^br^ subpopulation in the doxorubicin-treated group was not greater than that of the control group, and (3) The larger primary tumor volume in vehicle-treated control mice may influence the percentage of ALDH^br^ tumor cells due to tumor necrosis or hypoxia [64]. In our study, it was demonstrated for the first time that the frequency of ALDH^br^ tumor cells increased with the development of primary tumors in 4T1 tumor-bearing mice.

Although our oncolytic HSV1 can reduce the primary 4T1 tumor volume effectively, which is consistent with the previous reports [27,59,60,65,66], and decrease the frequency of ALDH^br^ cells compared with chemotherapy, it also has its own limitations, including physical barriers such as the extracellular matrix, which restrict the initial distribution and subsequent spread of viruses in the tumor mass when the oncolytic virus is directly injected into the tumor, and anti-HSV1 immunity, which can limit virus replication when locally or systematically given repeatedly [67,68]. These limitations may be overcome by combination of viral and chemotherapies. Ideally, the combined therapies could lead to synergistic effects in the following considerations: (1) The majority of the non-CSCs were first eradicated by chemotherapy and then the residual CSCs were killed by oncolytic HSV1. (2) Oncolytic viral replication and subsequent spread in the tumor mass may be enhanced by chemotherapy leading to antiviral immune response inhibition, the destruction of physical barriers, alteration in the tumor cell physiology and induced activation of DNA repair pathways [34,69,70]. (3) Oncolytic viruses circumvent typical chemoresistance mechanisms, they may be effective for chemoresistant CSCs [22,71]. (4) Chemotherapy and oncolytic HSV1 with different mechanisms of action could synergistically act to kill cancer cells and thus may achieve much more efficient antitumor activity [70,72]. In our *in vivo* study, treatment with doxorubicin chemotherapy followed by oncolytic HSV1 achieved more significant benefits than either single agent alone. (5) Oncolytic HSV1 had slight and different toxicities compared with chemotherapy [73]. The combination of the two modalities may minimize toxic side effects [70,74]. In our experiment, the administration of oncolytic HSV1-hGM-CSF alone was well tolerated, and administering HSV1-hGM-CSF followed by doxorubicin did not enhance the toxicity of the latter (Additional file 1: Figure S1). The strategy of targeting CSCs with oncolytic HSV1-hGM-CSF in combination with standard chemotherapy that kills non-CSCs may be applied to the treatment of human breast cancer in the clinic.

## Conclusion

In conclusion, this study suggested that 4T1 ALDH^br^ cells possess CSC characteristics. These ALDH^br^ cells were doxorubicin-resistant but still treatable to oncolytic HSV1. The treatment of 4T1 breast tumors with oncolytic HSV1 followed by doxorubicin chemotherapy generated a potent anticancer effect *in vivo.*

## Abbreviations

CSCs: Cancer stem cells; ALDH: Aldehyde dehydrogenase; OVs: Oncolytic viruses; HSV: Herpes simplex virus; MOI: Multiplicity of infection; GFP: Green fluorescent protein; hGM-CSF: human granulocyte-macrophage colony stimulating factor; PFU: Plaque forming units; CPE: Cytopathic effect; ND: Not determined; DEAB: Diethylaminobenzaldehyde; P-gp: P-glycoprotein; FCS: Fetal calf serum; FGM: Full growth medium; EGF: Human recombinant epidermal growth factor; bFGF: Human recombinant basic fibroblast growth factor; CMC: Carboxymethyl cellulose; PBS: Phosphate-buffered saline; SEM: Standard error of the mean; SDS-PAGE: Sodium dodecyl sulfate– polyacrylamide gel electrophoresis.

## Competing interests

The authors declare that they have no competing interests.

## Authors’ contributions

XFZ participated in the study design and carried out all experiments. She also performed the statistical analyses and drafted the manuscript. WZ assisted with flow cytometry sorting and western blot assays. YTC and XPH participated in dissection of mice and sample collection. JL and YZ assisted with experimental materials preparation. YHZ, SRZ and BLL conceived of the study, and participated in its design and helped to revise the manuscript. All authors read and approved the final manuscript.

## Pre-publication history

The pre-publication history for this paper can be accessed here:

http://www.biomedcentral.com/1471-2407/12/549/prepub

## Supplementary Material

Additional file 1 **Figure S1. **Toxicity comparison of four different treatment groups *in vivo.* Click here for file
