# Supplementary material for: Doxorubicin-enriched, ALDHbr mouse breast cancer stem cells are treatable to oncolytic herpes simplex virus type 1
Source: BMC Cancer. 2012 Nov 23;12:549. doi: 10.1186/1471-2407-12-549 (PMC3541265; doi:10.1186/1471-2407-12-549)
Supplement: Additional file 1 — Figure S1. Toxicity comparison of four different treatment groups in vivo. [file 1471-2407-12-549-S1.doc]

**Results**

**Oncolytic HSV1-hGM-CSF showed little side effect and would not enhance toxicity of doxorubicin**

Since treatment with doxorubicin plus HSV1-hGM-CSF manifested best antitumor potential, we then determined whether HSV1-hGM-CSF would enhance the toxicity of doxorubicin as evaluated by body weight loss, WBC numbers.

At the beginning of the study, the body weight of the mice in the four groups did not differ, and it was around 18 g/mouse (Additional Figure 1A). At the days 4 and 8 after treatment, the doxorubicin-treated group showed more body weight reduction compared with vehicle-treated control group or HSV1-hGM-CSF-treated group. Body weight between the two groups treated with doxorubicin plus HSV1-hGM-CSF and doxorubicin alone had not significant different (p>0.05). Almost no body weight reduction was shown in HSV1-hGM-CSF-treated groups. However, such drug-induced body weight lose was transient, body weights at the end of the study did not varied significantly between the four treatment groups.

To evaluate the myelotoxic effect of doxorubicin, blood was drawn from the eyes of mice and white blood cells (WBC) were counted on day 5 after twice doxorubicin chemotherapy and/or twice oncolytic HSV1-hGM-CSF treatment. Our results demonstrated that blood samples of the two treatment groups containing doxorubicin chemotherapy both less than the other two groups (p<0.05) (Additional Figure 1B). However, WBC number from mice treated with doxorubicin plus HSV1-hGM-CSF were similar to that of doxorubicin alone-treated mice, though the former was slightly less than the latter, no significant difference was found ( n=3, p>0.05). The number of WBC in vehicle-treated control was significantly high, above the normal WBC number, maybe because of the influence of the bigger tumors in control mice. Furthermore, the number of RBC and PLT in the four groups did not show significant difference (n=3, p> 0.05) (data not shown).

Collectively, these results revealed that, despite its high antitumor potency, oncolytic HSV1-hGM-CSF showed no obvious side effect. Furthermore, doxorubicin chemotherapy followed by oncolytic HSV1-hGM-CSF treatment is probably not much more toxic than doxorubicin alone.

**Figure legend**

**Additional Figure 1. Toxicity comparison of four different treatment groups *in vivo*.**

(A) Body weight loss comparison among the four treatment groups. (B) WBC count comparison among the four treatment groups.

Abbreviations: DOX, doxorubicin; OV, HSV1-hGM-CSF.


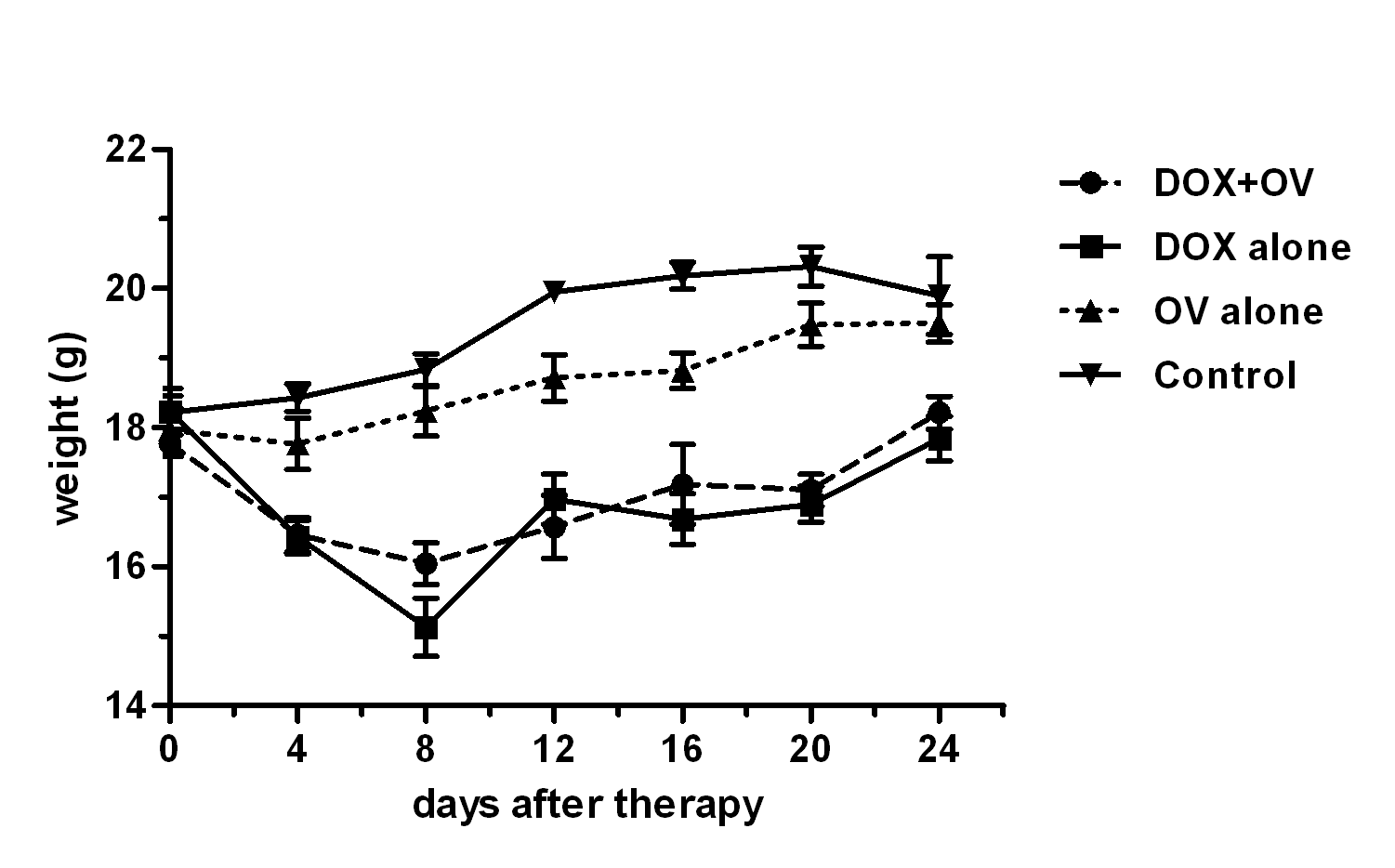


**A**

**B**


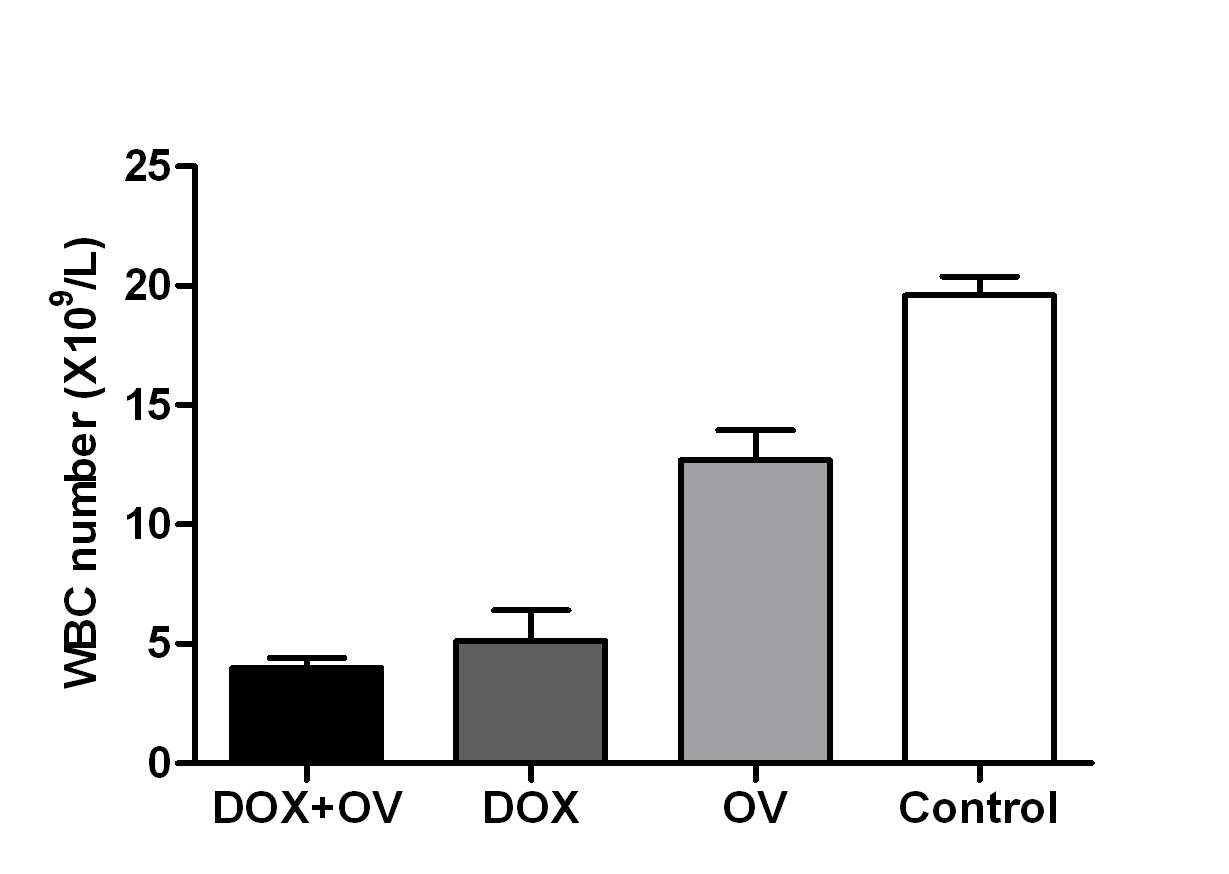


**Additional Figure 1**
